# Case Report: Recurrent Left Posterior Fascicular Ventricular Tachycardia in a Newborn

**DOI:** 10.3389/fped.2021.691146

**Published:** 2021-08-05

**Authors:** Lu Zhao, Lin Wu, Qu-ming Zhao, Xue-cun Liang

**Affiliations:** Department of Cardiology, Children's Hospital of Fudan University, Shanghai, China

**Keywords:** neonate, infant, arrhythmia, tachycardia, fascicular ventricular tachycardia

## Abstract

Left posterior fascicular ventricular tachycardia (LPFVT) is extremely rare in neonates. We described a 17-day-old girl with LPFVT who was initially misdiagnosed as supraventricular tachycardia (SVT). Eventually, she was successfully treated by amiodarone infusion followed by oral amiodarone with propranolol for 9 months, and LPFVT spontaneously resolved after a 1-year follow-up. This case report illustrated the basic principles and caveats in differential diagnosis of LPFVT in the neonatal age group. With proper diagnosis and therapy, neonatal LPFVT might regress in the first year of life.

## Introduction

Left posterior fascicular ventricular tachycardia (LPFVT) is extremely rare in newborns. To the best of our knowledge, only three neonatal cases have been reported in the literature since LPFVT was firstly described by Belhassen et al. in 1981 (1–4). LPFVT is frequently misdiagnosed as supraventricular tachycardia (SVT) in clinical practice because the QRS duration is slightly prolonged during tachycardia, particularly in infants. In this case report, we described a 17-day-old girl with recurrent LPFVT, focusing on the electrocardiogram (ECG) recognition, management, and prognosis of this type of arrhythmia in neonatal age group.

## Case Presentation

**Figure 1:**
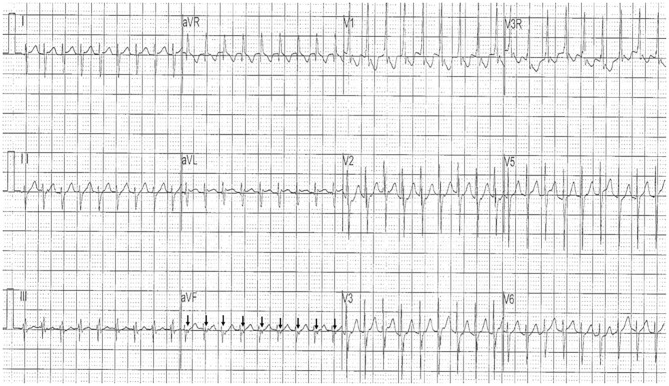
The initial 12-lead electrocardiogram showed regular tachycardia with a heart rate of 210 bpm, a slightly wide QRS duration of 72 ms, a superior QRS axis of 223°, and a right bundle branch block (RBBB) pattern. Reverse P′ waves (arrow) were identified in lead aVF, indicating the presence of 1:1 ventriculoatrial (VA) retrograde conduction.

**Figure 2:**
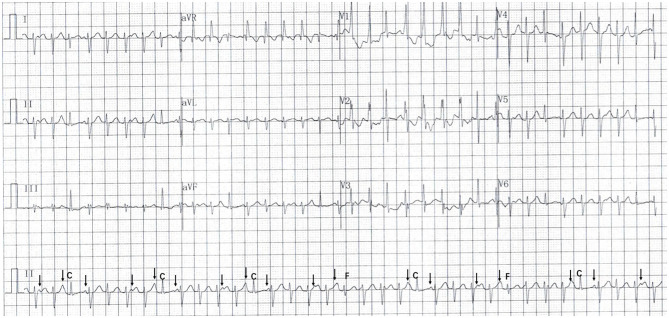
The electrocardiogram following amiodarone infusion revealed atrioventricular (AV) dissociation (arrow), capture beats (C), and fusion beats (F).

**Figure 3:**
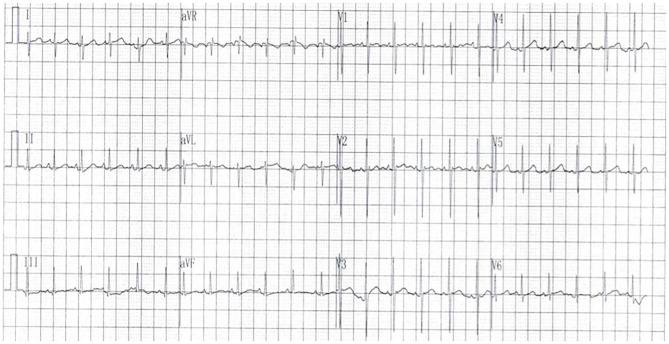
The 12-lead electrocardiogram showed sinus rhythm with a normal QRS duration of 62 ms, and a QRS axis of 72°.

A premature female newborn was delivered at a gestational age of 35^+4^ weeks in a local hospital, with birth weight of 2,410 g. She was transferred to NICU in our institution. On the 17th day after birth, tachycardia was identified by the monitor. A 12-lead electrocardiogram (ECG) demonstrated a regular tachycardia with a heart rate of 210 beats per minute (bpm), a slightly wide QRS duration of 72 ms, a superior QRS axis of 223°, and a right bundle branch block pattern (RBBB) (Figure 1). She was initially presumed to have SVT. The echocardiography showed a structurally normal heart with a patent foramen ovale and normal cardiac function. Ice dunking followed by three boluses of adenosine triphosphate (ATP) injection to a maximum dose of 0.5 mg/kg was unsuccessful in terminating the tachycardia. Thereafter, electrical cardioversion was tried and failed to revert the rhythm to sinus. She remained hemodynamically stable, and intravenous amiodarone infusion at a full dose of 15 mcg/kg/min was started. During continuous amiodarone infusion, ECG revealed atrioventricular (AV) dissociation, occasional capture beats, and fusion beats (Figure 2). The sinus rhythm with normal AV conduction and narrow QRS duration of 62 ms was achieved 6 h later (Figure 3). By then, a diagnosis of LPFVT was established by reviewing all ECG strips. Non-sustained tachycardia recurred when intravenous amiodarone was gradually weaned off. The patient was discharged on oral amiodarone (5 mg/kg/day) with propranolol (2 mg/kg/day), despite that non-sustained episodes of LPFVT were documented by Holter. At 2 months of age, the girl was admitted to the emergency room for sustained LPFVT over 1 h. Tachycardia was successfully terminated by electrical cardioversion, and the medication dose for prophylaxis arrhythmia was increased proportionally to her weight increment. She was closely followed up by Holter every 4–8 weeks, and there was no recurrence of LPFVT after 6 months. Anti-arrhythmia medications were discontinued at 9 months. Her liver function and thyroid function were assessed every 3 months prior to discontinuation of medication and were within the normal range. Her last Holter evaluation at 13 months of age was completely normal. The timeline of diagnosis and therapy of LPFVT in this neonatal case are set out in Table 1.

**Table 1:** The timeline of patient diagnosis and therapy.

**Age**	**Event**	**Management**
Day 0	Premature delivery	Transferred to our institution
Day 17	Tachycardia	Diagnosis of SVT was presumed Ice dunking, ATP injection, and electrical cardioversion failed to terminate tachycardia Intravenous amiodarone was started
Day 18	Termination of sustained tachycardia	ECG revealed AV dissociation, capture beats and fusion beats. Diagnosis was revised to LPFVT
Day 18–33	Non-sustained tachycardia	Gradual withdrawal of amiodarone infusion
Day 33	Non-sustained tachycardia	Oral propranolol (2 mg/kg/d) and amiodarone (5 mg/kg/d) were started
Day 42	Non-sustained tachycardia	Discharged
2 months	Sustained tachycardia over 1 hour	Electric cardioversion reverted LPFVT to sinus rhythm Increased dose of propranolol (2 mg/kg/d) and amiodarone (5 mg/kg/d) proportionally to her weight increment
3–5 months	Non-sustained tachycardia	Holter followed up every 4–8 weeks
6 months	Sinus rhythm	Oral propranolol and amiodarone continued
9 months	Sinus rhythm	Oral propranolol and amiodarone discontinued
13 months	Sinus rhythm	Last Holter followed up

*SVT, supraventricular tachycardia; ATP, adenosine triphosphate; ECG, electrocardiogram; AV, atrioventricular; LPFVT, left posterior fascicular ventricular tachycardia*.

## Discussion

Fascicular ventricular tachycardia (VT) is a form of idiopathic and Purkinje-related arrhythmia and is rarely reported in newborns. LPFVT is the most common form of fascicular VT, originating in the left posterior fascicle (5, 6).

The typical ECG pattern of LPFVT is characterized by right bundle branch block and superior axis deviation. The QRS morphology is mildly widened during tachycardia. It is often misdiagnosed as SVT with RBBB and left anterior hemiblock aberrancy. In the published literatures, the initial misdiagnosis rate of LPFVT in young infants is as high as 40% (2–4, 7–11). Therefore, we would like to demonstrate the basic principles and caveats in differential diagnosis of LPFVT in the neonatal age group by reviewing the present case. Firstly, VT in neonates is rare but does occur. It is important to bear in mind that the upper limit of normal QRS duration is 65 ms in neonates (12). In the present case, QRS duration during tachycardia was 72 ms, which exceeded the normal range. The diagnosis of VT should therefore be assumed. Secondly, AV dissociation, capture beats, and fusion beats during tachycardia are diagnostic markers of VT, but these studies are not usually present in neonates due to the rapid ventricular rate and presence of ventriculoatrial (VA) retrograde conduction. In reviewing the initial ECG strip of this case, the reverse P′ wave during VT was recognized, which indicated VA retrograde conduction. AV dissociation, capture beats, and fusion beats were identified during amiodarone infusion. Thirdly, LPFVT does not respond to adenosine (2, 8, 13), while other types of wide QRS tachycardia, which need to be differentiated from VT, including SVT with bundle branch block and SVT with anterograde conduction across an accessory pathway, can usually be terminated by adenosine. Thus, if adenosine injection is not effective in reverting presumed SVT, possibility of LPFVT should be considered.

The experience in management of infantile LPFVT is very limited. Verapamil is most commonly used in older children and has been shown to terminate tachycardia in 93% of children with LPFVT (13). However, its use in infants is widely considered to be contraindicated due to the risk of hemodynamic collapse (14). Beta blockers, amiodarone, and electrical cardioversion are usually recommended. Electrical cardioversion could achieve termination of LPFVT in 62% of children, and the effective rate of amiodarone infusion is not clearly known (13–15). In our neonatal case, LPFVT did not respond to electrical cardioversion, while it was reverted to sinus rhythm by amiodarone infusion. Oral amiodarone with propranolol had been given for 9 months to prevent recurrence until LPFVT regressed. Verapamil has recently been used for arrhythmia in infants (2, 16, 17). Slow intravenous infusion at a dose of 0.1 mg/kg over at least 10 min appeared to be safe and efficient in terminating infantile LPFVT, but only one newborn was enrolled (2, 7, 9, 11).

LPFVT is often considered to be a type of idiopathic VT with good prognosis, but delayed diagnosis and therapy can result in severe cardiac dysfunction (8). The eventual resolution of LPFVT in our neonatal case is consistent with the previous report by Collins et al., which showed that LPFVT occurring in the first year of life may resolve spontaneously (13).

## Conclusion

This illustrative case report describes a neonate with LPFVT that was initially misdiagnosed as supraventricular tachycardia. The clinical course highlights the caveats of differential diagnosis in neonatal ECG evaluation and the favorable outcome of LPFVT. With proper diagnosis and treatment, LPFVT may regress over time within the first year of life.

## Data Availability Statement

The original contributions presented in the study are included in the article/supplementary material, further inquiries can be directed to the corresponding author/s.

## Ethics Statement

Written informed consent was obtained from the minor(s)' legal guardian/next of kin for the publication of any potentially identifiable images or data included in this article.

## Author Contributions

All authors listed have made a substantial, direct and intellectual contribution to the work, and approved it for publication.

## Conflict of Interest

The authors declare that the research was conducted in the absence of any commercial or financial relationships that could be construed as a potential conflict of interest.

## Publisher's Note

All claims expressed in this article are solely those of the authors and do not necessarily represent those of their affiliated organizations, or those of the publisher, the editors and the reviewers. Any product that may be evaluated in this article, or claim that may be made by its manufacturer, is not guaranteed or endorsed by the publisher.
